# Secondary Somatic Embryogenesis in *Centaurium erythraea* Rafn

**DOI:** 10.3390/plants10020199

**Published:** 2021-01-21

**Authors:** Milica D. Bogdanović, Katarina B. Ćuković, Angelina R. Subotić, Milan B. Dragićević, Ana D. Simonović, Biljana K. Filipović, Slađana I. Todorović

**Affiliations:** Department of Plant Physiology, Institute for Biological Research “Siniša Stanković”-National Institute of Republic of Serbia, University of Belgrade, Bulevar Despota Stefana 142, 11060 Belgrade, Serbia; katarina.cukovic@ibiss.bg.ac.rs (K.B.Ć.); heroina@ibiss.bg.ac.rs (A.R.S.); mdragicevic@ibiss.bg.ac.rs (M.B.D.); ana.simonovic@ibiss.bg.ac.rs (A.D.S.); biljana.nikolic@ibiss.bg.ac.rs (B.K.F.); slatod@ibiss.bg.ac.rs (S.I.T.)

**Keywords:** cyclic somatic embryogenesis, direct somatic embryogenesis, indirect somatic embryogenesis, leaf explant, histology, 2,4-D, CPPU, auxins, cytokinins

## Abstract

Somatic embryogenesis (SE) is a developmental process during which plant somatic cells, under suitable conditions, produce embryogenic cells that develop into somatic embryos (**se**). SE is the most important method for plant propagation in vitro, having both fundamental and applicative significance. SE can be induced from different tissues and organs, but when **se** are used as explants, the process is recognized as secondary or cyclic SE. We induced secondary SE in *Centaurium erythraea* by application of 2,4-dichlorophenoxyacetic acid (2,4-D) and *N*-(2-chloro-4-pyridyl)-*N*′-phenylurea (CPPU). A medium containing 0.1 mgL^−1^ 2,4-D and 0.25 mgL^−1^ CPPU was optimal in terms of the number of primary SE explants forming **se**, the number of well-developed **se** per explant, and morphological appearance of the obtained **se**. These concentrations allowed SE to progress through three cycles, whereas at higher concentrations of 0.2 mgL^−1^ 2,4-D and 0.5 mgL^−1^ CPPU, only two cycles were achieved. Histological analysis revealed that secondary **se** are formed both directly and indirectly. Secondary SE readily germinated and converted into plantlets. Induction of cyclic SE contributes to the conservation efforts of this endangered medicinal plant and expands the spectrum of in vitro developmental pathways described in centaury—an emerging model in developmental biology.

## 1. Introduction

*Centaurium erythraea* Rafn (common, European, or small centaury), belonging to the Gentianaceae family, is a medicinal plant with a broad environmental tolerance. Centaury is widespread over most of Europe, where it grows in different habitats, such as on river banks and wood margins, as well as on calcareous dry and sandy land [1,2]. The aerial part of the plant, *Centaurii herba*, is traditionally used as bitter tinctures, tonics, lotions, or teas to treat a diversity of ailments. The bitter taste is due to the secoiridoids. Secoiridoid glucosides are reported in various applications for the treatment of different digestive problems, as well as gastroprotective [3] and hepatoprotective agents [4]. Other important secondary metabolites include xanthones [5], as well as alkaloids, terpenoids, phenolic acids, flavonoids, fatty acids, alkanes, and waxes [6,7], some of which are constituents of centaury essential oils [8]. Centaury also exhibits considerable antioxidant [7,9,10], antidiabetic [11,12], and antimicrobial [7] pharmacological properties, which are correlated with its phytochemical composition. Because of the extensive and uncontrolled exploitation, coupled with its limited cultivation restricted by unpredictable seed germination and the inability of *C. erythraea* to grow in dense stands [6], as well as insufficient attempts for the replenishment, the wild populations of centaury have been markedly depleted. Sustainable utilization of this valuable medicinal plant and the efforts for its conservation, as well as biotechnological alternatives for the production of its secondary metabolites, rely on the development of efficient in vitro techniques for the mass propagation of centaury [13]. As reviewed in the accompanying article in this issue [14], the most extensively studied pathway of centaury in vitro propagation is somatic embryogenesis (SE).

SE is a developmental process by which plant somatic cells, under suitable inductive conditions, produce embryogenic cells that, through a series of morphological and biochemical changes, form a somatic embryo [15]. Somatic embryo (**se**) is a structure that resembles the zygotic embryo, but formed without fertilization, which passes through similar stages (globular, heart-shaped, torpedo-shaped, and cotyledonary embryo). As such, **se** is not enclosed by maternal tissues, so that the process of SE can be not only controlled by the in vitro culture conditions, but the obtained **se** can be observed and collected at different developmental stages for the molecular and biochemical analyses [15]. Thus, SE is used as a model system for studying morphological, physiological, and molecular aspects of embryogenesis in higher plants [15,16], as well as for investigating cellular differentiation and mechanisms leading to acquisition of totipotency in plant cells [17]. Equally important are various biotechnological applications of SE, such as the propagation of elite or transgenic lines, while single-cell origin of some **se** may offer many advantages for breeding programs [18]. Actually, SE is considered as the most appropriate in vitro method for the clonal propagation of different plant species due to its high multiplication potential [19]. In addition, plant propagation trough SE represents an important source of material for plant transformation, offering genetically identical starting material, with less somaclonal variation as compared to propagation through organogenesis [20]. SE has also been revealed as the best regeneration pathway in cryopreservation [21], as well as a method of choice for the haploid production, somatic hybridization, and the production of artificial seeds [16].

Somatic embryos can either differentiate directly, from a small group of cells of the explanted tissue (direct SE or DSE), or indirectly, from embryogenic callus cells that further produce embryos (indirect SE or ISE) [22]. SE is influenced by internal and external factors, such as the type and the physiological state of the explant, the composition of the culture medium, the type and concentration of plant growth regulators (PGRs) in the medium, temperature, and light regime [23]. It has been suggested that in the case of DSE, proembryogenic competent cells are already present in the explant, hence they require minimal reprogramming, while in ISE, major cell reprogramming is needed to acquire embryogenic potential [24].

In most plant species, particularly in the Gentianaceae family, PGRs in the auxin and cytokinin groups are among the main factors affecting the induction of SE [25]. They determine the acquisition of totipotency by the explant cells [26], and consequently induce the development of **se**. Auxins and cytokinins are involved in the regulation of cell divisions and differentiation processes in the plant tissues [27], leading to the formation of SE. In the protocols for the induction of SE in many plant species, cell reprogramming is induced by a treatment with exogenous auxin, usually 2,4-dichlorophenoxyacetic acid (2,4-D). The evidence supports the notion that auxins play a critical role in cell reprogramming, while the induction of SE development requires subsequent elimination of the auxin from the culture media [28]. The concentrations of auxin required for SE induction may vary with different protocols. Cytokinins are thought to be more involved in the differentiation and further development of SE. In most plant species, the SE induction requires the presence of both PGRs [29], but SE can also be achieved by using only certain auxins [30] or cytokinins [31].

Secondary SE is a developmental process by which new (secondary) somatic embryos are developed from the primary somatic embryos used as explants. Other common names for secondary SE are repetitive, cyclic, recurrent, accessory, or adventitious SE. Secondary **se** are formed directly or/and indirectly on the cotyledons, hypocotyls, epicotyls, or root tips of the primary **se**. Efficient plant regeneration systems through secondary SE have been reported in several plant species, for example in *Cyclamen persicum* [32], *Hepatica nobilis* [33], *Pseudotsuga menziesii* [34], *Akebia trifoliata* [35], and *Olea europea* [36]. Secondary SE may enhance and prolong embryogenic competence of certain lines [34], multiply the number of embryos that can be obtained (compared to primary SE) [34], and recycle **se** of abnormal morphology that otherwise cannot regenerate normal plants [37]. This developmental pathway characterizes high multiplication index, repeatability, independence from explants source effects, and high level of uniformity [38]. Low production rate of important clone lines can be enhanced by obtaining secondary SE [34]. Since in many species, embryogenic competence in the in vitro culture declines over time due to aging and subculturing for several months [39,40], secondary SE provides a way to restore the embryogenic potential of important productive lines and is routinely used with broadleaved tree species as a method of long-term management [34].

As discussed in detail in the accompanying review article of this issue [14], several successful protocols for the induction of SE in *C. erythraea* from different explants have been published. Briefly, SE has been induced in cell suspension culture [41], root explants [6,42,43,44,45], and leaf explants [13,46]. While SE from roots was spontaneous and direct, the SE from the leaf explants was indirect and induced by the addition of 2,4-D and a urea-type synthetic cytokinin *N*-(2-chloro-4-pyridyl)-*N*′-phenylurea (CPPU). There are no reports on the induction of secondary SE in centaury. Hereby, we report a successful establishment of secondary SE in centaury, as a valuable addition to the spectrum of protocols for the in vitro propagation of this species. Namely, we have recently proposed *C. erythraea* as a model plant organism in developmental biology due to its great regeneration potential and developmental plasticity [46,47]. So, the present work aims not only to provide a more efficient way for the in vitro propagation of centaury as a support for the conservation efforts, but also to establish an additional system for fundamental research of centaury development. Namely, having several systems for the induction of SE from different explants in the same species would allow for a comparison of biochemical and molecular events in these systems within the same genetic background. The effects of different combinations of 2,4-D and CPPU on the induction of SE are described, along with the effects of multiple cycles of SE on the efficiency of this process. The results are supported by histological analyzes of the embryogenic tissues and developing **se**, as well as the germination tests of the obtained secondary **se**.

## 2. Results

### 2.1. Induction of Primary SE from C. erythraea Leaf Explants and the Experimental Setup

**Figure 1:**
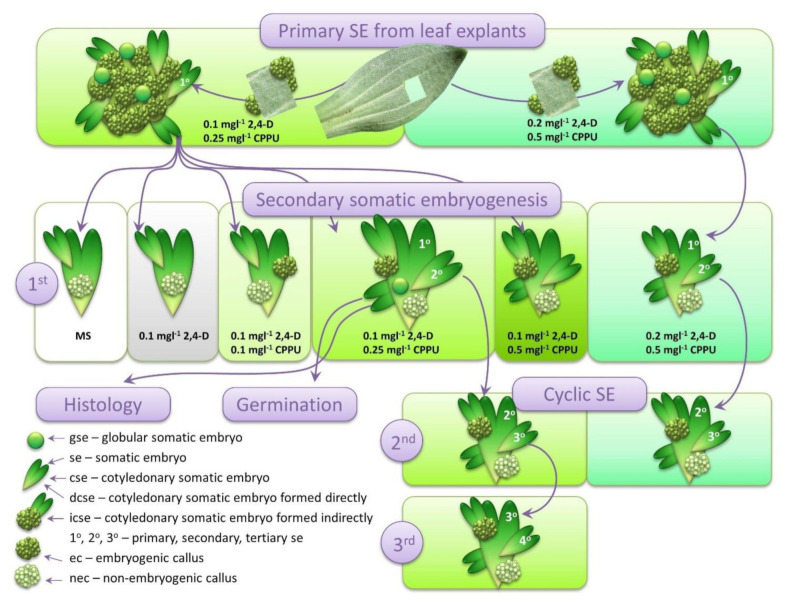
Schematic presentation of the experimental setup. Primary cotyledonary somatic embryos (**cse**) developed on the centaury leaf explants provided the initial explants for the induction of secondary somatic embryos (SE). The induction of cyclic SE was conducted on two different media. Secondary embryos developed on 0.1 mgL^−1^ 2,4-dichlorophenoxyacetic acid (2,4-D) and 0.25 mgL^−1^ *N*-(2-chloro-4-pyridyl)-*N*′-phenylurea (CPPU) were examined histologically and their germination was tested. Rectangles of identical colors represent the same composition of the culture medium. The listed abbreviations are used throughout the text.

**Figure 2:**
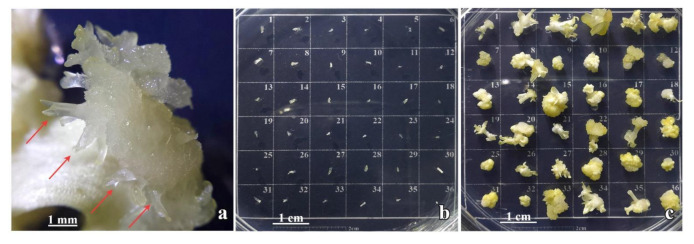
The induction of secondary SE in *Centaurium erythraea.* (**a**) Primary **cse** developed on leaf explants (red arrows); (**b**) primary **cse** were excised and set as explants arranged as 6 × 6 array on MS medium containing 0.1 mgL^−1^ 2,4-D and 0.25 mgL^−1^ CPPU; (**c**) primary **cse** with induced secondary embryos.

We have previously published successful induction of SE from centaury leaf explants cultivated on a combination of 2,4-D and CPPU, where ISE proceeds as a sole developmental pathway, providing that the leaf explants are kept in darkness [13]. Thus, primary SE was induced as described by Filipović et al. [13], with slight modifications. The leaf segments of mature plants were cultivated for three weeks in the darkness on MS medium supplemented with 0.1 mgL^−1^ 2,4-D and 0.25 mgL^−1^ CPPU (Figure 1). Well-developed primary cotyledonary somatic embryos (**cse**) (Figure 1 and Figure 2a) formed on this media were used as primary explants for the induction of secondary SE on media with varying 2,4-D and CPPU concentrations (Figure 1). Generally, in all experiments described below, only **cse** were used as explants, even though embryos of all developmental stages were observable. For a comparison, the leaf explants were also cultivated on a media supplemented with 0.2 mgL^−1^ 2,4-D and 0.5 mgL^−1^ CPPU, and the primary **cse** obtained in this setup were also used for the induction of secondary and cyclic SE, as discussed later.

### 2.2. Induction of Secondary SE

**Figure 3:**
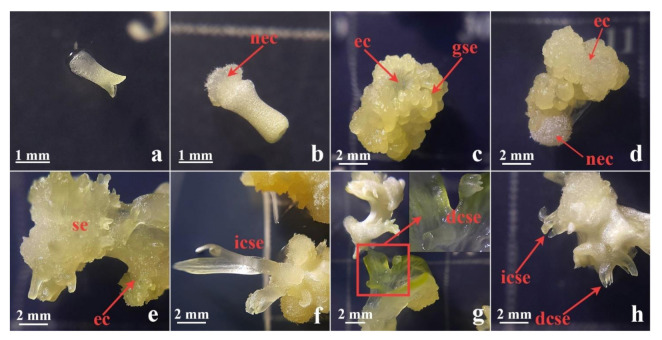
Secondary SE after four weeks in culture on medium with 0.1 mgL^−1^ 2,4-D and 0.25 mgL^−1^ CPPU. (**a**) Primary explant—**cse** at the beginning of the experiment; (**b**) **cse** with only nonembryogenic calli (**nec**) developed; (**c**) **cse** with developed embryogenic calli (**ec**) and globular somatic embryo (**gse**); (**d**) **cse** that developed both **ec** and **nec**; (**e**) **ec** with secondary **se** of various developmental stages; (**f**) cotyledonary somatic embryo formed indirectly (**icse**) developed from callus; (**g**) cotyledonary somatic embryo formed directly (**dcse**) developed on cotyledons of primary **cse** explants; (**h**) both **dcse** and **icse** types of embryos on the same explant. **cse**—cotyledonary somatic embryo, **nec**—nonembryonic callus, **ec**—embryogenic callus, **gse**—globular somatic embryo, **dsce**—cotyledonary somatic embryo formed directly, **icse**—cotyledonary somatic embryo formed indirectly.

Well-developed primary **cse** formed on the leaf explants on 0.1 mgL^−1^ 2,4-D and 0.25 mgL^−1^ CPPU (Figure 2a) were excised (Figure 2b and Figure 3a) and used as explants for the induction of secondary SE on the same medium. After four weeks, primary **cse** explants enlarged several times and developed both embryogenic calli (**ec**) and nonembryogenic calli (**nec**), as well as somatic embryos at different developmental stages (Figure 2c). The **nec** that developed on primary **cse** was watery, friable, and translucent (Figure 3b). In contrast, the **ec** exhibited more organized structure and morphology: the embryogenic tissue was semi-compact, nodular, with a smooth surface and whitish to yellowish color (Figure 3c–e). Secondary **se** developed on the primary **cse** both indirectly, from the **ec** (Figure 3e,f,h), as well as directly on the explants, without callusing (Figure 3g,h). Somatic embryos at the cotyledonary stage formed directly or indirectly are referred to in the following text as **dcse** and **icse**, respectively. Although most of the explants swelled and significantly changed their morphology during the cultivation, in some of the explants, **dcse** could be observed developing on the primary cotyledons (Figure 3g).

### 2.3. Histology of the Secondary SE

**Figure 4:**
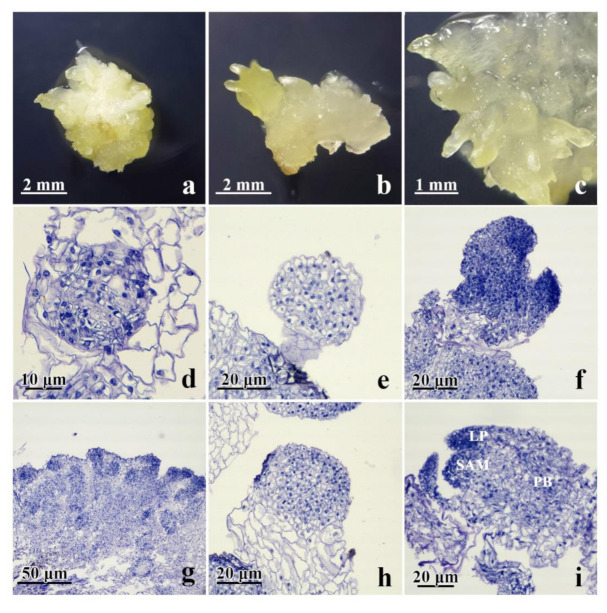
Morphological and histological characterization of secondary SE from primary **cse** explants cultured for 3 weeks in the darkness on medium supplemented with 0.1 mgL^−1^ 2,4-D and 0.25 mgL^−1^ CPPU. (**a**–**c**) Numerous secondary **cse** were visible to arise indirectly from **ec** and/or directly from the primary explant; (**d**–**f**) the process of secondary DSE; (**g**–**i**) the process of secondary ISE; (**d**) developed proembryogenic cell masses (PEMs); (**e**) formation of secondary **gse** with a suspensor-like structure at the surface of primary **cse**; (**f**) secondary **cse** at early stage of development; (**g**) embryogenic callus formed of small and isodiametric clumps of cells; (**h**) globular somatic embryo formed from **cse**; (**i**) secondary **cse** at a late developmental stage with visible shoot apical meristem (SAM), provascular bands (PB), and leaf primordia (LP).

The process of the development of secondary somatic embryos in centaury was histologically analyzed. As explained in the previous section, secondary SE was induced on media containing 0.1 mgL^−1^ 2,4-D and 0.25 mgL^−1^ CPPU. Morphological features of the explants with directly and/or indirectly developed secondary **se** at different stages, that were used for the histological analysis, are shown in Figure 4a–c. The presence of the **se** at different developmental stages that could be observed on the same explant suggests that the process of SE is asynchronous. The major events during the SE are comprised of early, intermediate, and the maturation stages. Early stages of SE are described as the process of the induction of **ec**, as well as induction of direct **se** from the subepidermal cells of the explant. This is followed by the intermediate stage of SE, during which **se** are initiated either indirectly, from the proembryogenic masses (PEM), or directly, following the activation of repeated cell divisions of the proembryogenic cells. Finally, the maturation stage of SE is the final stage of vascular patterning for the induction of shoot apical meristem (SEM), leaf primordia, and provascular bands. Histological features observed during DSE from centaury roots [42,43] and ISE from the leaf explants [13] aid in distinguishing these two types of SE, which occur simultaneously during secondary SE.

Direct induction of secondary **se** was seen from the entire surface of the primary **se**. The direct initiation of **se** was observed from the subepidermal layer of the **cse** explant without an intervening callus phase. Differentiated multicellular PEMs, seen at the periphery of the explants, further developed into embryos (Figure 4d). During further growth, the PEM produced secondary somatic embryos at the globular stages of development (**gse**). The **gse** at the onset of polarization, showing a protoderm-like layer, were the first clearly distinguishable stage of the somatic embryo differentiation (Figure 4e). These **gse** had no apparent vascular connection with the primary **cse** tissue to which they are connected by a suspensor-like structure (Figure 4e). Subsequent somatic embryo development included their elongation, development of procambium, and shoot meristem differentiation, reaching the early cotyledonary-staged somatic embryo (Figure 4f). The secondary **cse** were poorly attached to the surface of the primary explants and could easily separate. No vascular connections were observed between the developing somatic embryos and the primary explant (Figure 4f).

As observed using light microscopy, the **ec** was formed by small and isodiametric clumps of cells, containing prominent nuclei and dense cytoplasm. These clusters were round structures bounded by a layer of organized cells. Histological analyses showed that some cells in the superficial portions of this callus exhibited meristematic characteristics (Figure 4g). These clusters of proembryogenic cells progressed through a series of organized division to give rise to **gse** (Figure 4h). Finally, an increased embryo differentiation and elongation were observed, which became sharper, reaching the late **cse** with well-developed shoot apical meristem, leaf primordia and provascular bands (Figure 4i).

### 2.4. The Effect of 2,4-D and CPPU on the Induction of Secondary SE

**Figure 5:**
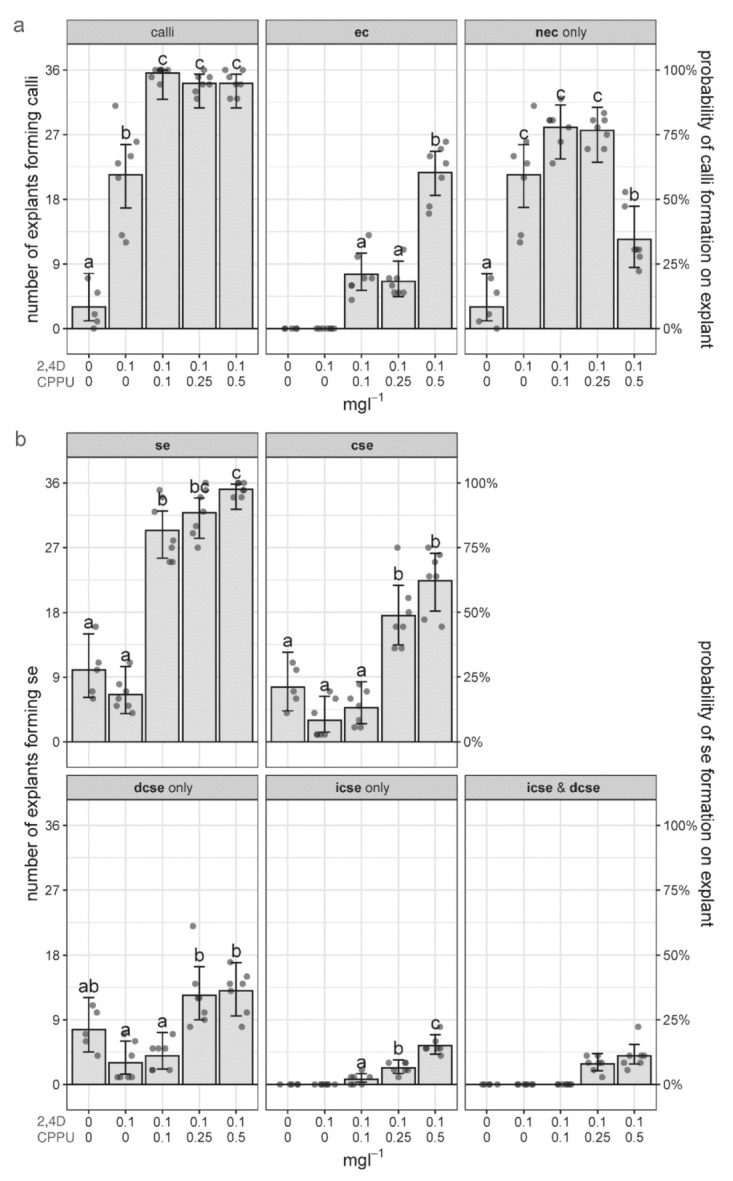
The effect of different concentrations of 2,4-D and CPPU on the induction of calli and secondary embryos on the **cse** explants. (**a**) The number of **cse** explants forming any type of calli and specifically embryogenic (**ec**) or nonembryogenic (**nec**) calli; (**b**) the number of **cse** explants forming secondary embryos of any stage or origin (**se**) and specifically cotyledonary somatic embryos (**cse**), formed directly (**dcse**) or indirectly (**icse**). The mean values for seven replicates (with 36 **cse** explants each) equivalent to fitted values of the corresponding logistic regression models, with error bars (95% confidence intervals) are presented as the number of explants forming specific types of calli or SE (left ordinate), or the probability of calli or SE formation on the explants (right ordinate). Different letters denote statistically significant differences at *p* < 0.05. Gray dots represent individual replicates (Petri dishes).

To explore the effect of different concentrations of 2,4-D and CPPU on the process of secondary SE, well-developed primary **cse** were excised from the leaf explants cultured on 0.1 mgL^−1^ 2,4-D and 0.25 mgL^−1^ CPPU and transferred to fresh media with different content of PGRs. The media were supplemented with 2,4-D (0.1 mgL^−1^) and increasing CPPU concentrations (0–0.5 mgL^−1^), whereas a hormone-free medium was used as control (Figure 1 and Figure 5). After 4 weeks in culture, each primary **cse** was observed, and features such as development of calli (**ec** or **nec**) and **se** at different developmental stages (cotyledonary or other) and of different origin (directly or indirectly formed) were recorded as a number of explants with a particular feature.

General regenerative potential of the **cse** explants on different media was initially evaluated as the number of explants forming calli. On a hormone-free medium, on average, only 3 out of 36 explants formed calli, exclusively nonembryogenic ones (**nec**), while no explants formed embryogenic (**ec**) calli (Figure 5a). In the **cse** explants cultivated on 0.1 mgL^−1^ 2,4-D, on average, about 60% of the explants produced calli, but again, all of them were **nec**. However, if both PGRs were added to the medium, almost all of the **cse** (on average 34.14–35.57 explants per treatment) generated calli, even at the lowest CPPU concentration of 0.1 mgL^−1^ (Figure 5a). Therefore, CPPU was necessary for the generation of **ec**. The concentration of 0.5 mgL^−1^ CPPU gave the highest number of explants with **ec** (21.71 on average), with concomitant reduction of the number of **cse** where only **nec** formed (Figure 5a).

**Figure 6:**
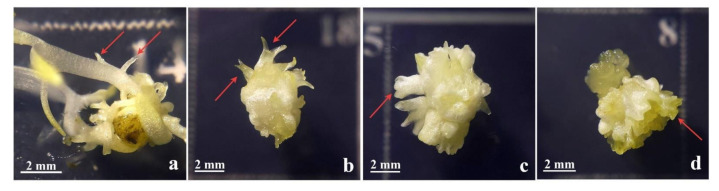
Morphology of secondary **cse** (red arrows) developing on different hormone combinations: (**a**) hormone-free medium; (**b**) 0.1 mgL^−1^ 2,4-D with 0.25 mgL^−1^ CPPU; (**c**) 0.1 mgL^−1^ 2,4-D with 0.5 mgL^−1^ CPPU; and (**d**) 0.2 mgL^−1^ 2,4-D with 0.5 mgL^−1^ CPPU.

Even though the primary explants cultivated on media without CPPU did not produce any embryogenic calli, secondary **se** did appear, on average, on 10 (out of 36) explants on the MS medium, and on 6.57 explants grown on 0.1 mgL^−1^ 2,4-D (Figure 5b). Obviously, in the absence of **ec**, all of the secondary **se** formed on these media were developed directly. Spontaneous secondary DSE is depicted in Figure 6a. With the addition of CPPU, the number of explants developing **se** drastically increased in a dose–response manner, up to, on average, 35.14 explants on the media supplemented with 0.5 mgL^−1^ CPPU (Figure 5b). Only **cse** were further classified into embryos formed directly and indirectly (**dcse** and **icse**), and it turned out that DSE is not only the exclusive path on CPPU-free media, but also the predominant path on media containing CPPU (Figure 5b). However, the number of explants with embryos developed by ISE slightly increased with rising the CPPU concentration, up to an average of 5.43 explants on medium with 0.1 mgL^−1^ 2,4-D and 0.5 mgL^−1^ CPPU (Figure 5b, “**icse** only”), in concordance with higher induction of **ec** on this medium (Figure 5a).

In addition, the number of **cse** per explant was also scored after 4 weeks of cultivation on different media (Table 1). The average number of the **cse** per explant was significantly higher on media containing 0.25 and 0.5 mgL^−1^ CPPU, as compared to other media, reaching up to 3.87 ± 0.50 new secondary **cse** per primary explant (Table 1).

The induction of secondary SE on different media produced **cse** of different morphology. On the hormone-free media, secondary **se** formed on the primary explants spontaneously and directly, mostly in the hypocotyl zone of the explant, and had normal morphology (Figure 6a). Concentration of 0.1 mgL^−1^ 2,4-D with 0.25 mgL^−1^ CPPU resulted in a production of well-developed bi-cotyledonary **se** (Figure 6b), which were easily isolated from the primary explant (and thus preferable as explants for new cycles of SE, as discussed later). Secondary **cse** forming on media formulations with higher CPPU concentration (0.5 mgL^−1^ CPPU) often had an abnormal morphology, with **cse** having more than two cotyledons, trumpet or fascicular shape, and fused cotyledons (Figure 6c,d).

### 2.5. Induction of Cyclic SE

To see whether SE can continue through several cycles, two combinations of PGRs were tested: 0.1 mgL^−1^ 2,4-D and 0.25 mgL^−1^ CPPU or 0.2 mgL^−1^ 2,4-D and 0.5 mgL^−1^ CPPU, referred in the following text as lower and higher concentrations, respectively. These two combinations of PGRs were maintained throughout this experiment, starting with the induction of SE on the leaf explants (see Figure 1). The four replicates (Petri dishes) that were used in this experiment were linked through the cycles in terms that the embryos developed in one cycle were transferred to a Petri dish with the same label in the next cycle. Since the highest number of the explants forming **se** or **cse** (Figure 5b), as well as the highest number of the **cse** per explant (Table 1), were obtained on 0.1 mgL^−1^ 2,4-D and 0.25 or 0.5 mgL^−1^ CPPU, the lower concentration of CPPU was used in combination with 2,4-D (0.1 mgL^−1^ 2,4-D and 0.25 mgL^−1^ CPPU) because the embryos developed on this media exhibited normal morphology (Figure 6b). On the other hand, the higher concentrations of both PGR but at the same ratio (0.2 mgL^−1^ 2,4-D with 0.5 mgL^−1^ CPPU) proved to be optimal for the induction of SE in centaury leaf culture [13], and for this reason were also used in this experiment. Only somatic embryos at the cotyledonary stage (**cse**) were harvested and set to initiate the next cycle.

**Figure 7:**
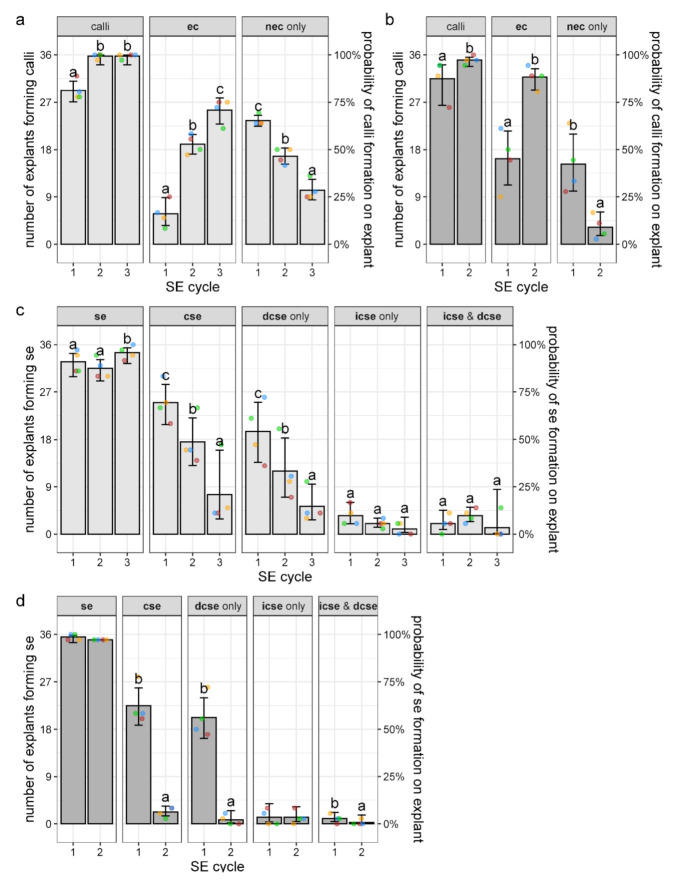
The effect of secondary SE cycle and media composition on the induction of calli and secondary embryos on explants. (**a**,**c**) The explants cultivated on lower concentrations of plant growth regulators (PGRs) (0.1 mgL^−1^ 2,4-D and 0.25 mgL^−1^ CPPU); (**b**,**d**) the explants are cultivated on higher concentrations of PGRs (0.2 mgL^−1^ 2,4-D and 0.5 mgL^−1^ CPPU); (**a**,**b**) the number of **cse** explants forming any type of calli and specifically embryogenic (**ec**) or nonembryogenic (**nec**) calli; (**c**,**d**) the number of **cse** explants forming secondary embryos of any stage or origin (**se**) and specifically cotyledonary somatic embryos (**cse**), formed directly (**dcse**) or indirectly (**icse**). The mean values for four replicates (with 36 **cse** explants each), equivalent to fitted values of the corresponding general estimating equations models, with error bars (95% confidence intervals), are presented as the number of explants forming specific types of calli or **se** (left ordinate), or the probability of calli or **se** formation on the explants (right ordinate). Dots of the same color represent linked replicates (Petri dishes) through the cycles. Different letters denote statistically significant differences at *p* < 0.05.

In the 1st cycle of the cyclic SE, secondary **cse** that developed on primary **cse** explants on media with lower or higher PGRs concentration were excised, transferred to a fresh media of the same composition, and their morphological features (developed calli and embryos) were scored after 4 weeks in culture. In the 2nd cycle, tertiary **cse** that formed after four weeks were subcultured on the same fresh media for the 3rd and last cycle (see Figure 1). As can be seen in Figure 7, both hormone combinations were able to initiate cyclic SE, but as discussed later, only SE on the lower concentrations could progress through three cycles, whereas only two cycles were possible on higher concentrations.

On the medium with lower PGRs, on average, 29.25 of the **cse** explants produced some type of calli during the 1st cycle, whereas nearly all explants (35.75 on average) generated calli during the 2nd and the 3rd cycle (Figure 7a). Likewise, the number of explants forming calli increased in the 2nd cycle, as compared to the 1st cycle, on higher PGRs concentration (Figure 7b). The embryogenic capacity of the explants measured as the number of **cse** explants producing embryogenic calli (**ec**) also significantly increased with the cycles’ progression on both types of media. Specifically, the number of explants forming **ec** on 0.1 mgL^−1^ 2,4-D and 0.25 mgL^−1^ CPPU increased through the cycles from 5.75 explants (per replicate) in the 1st cycle to 25.5 explants in the 3rd cycle (Figure 7a), while on 0.2 mgL^−1^ 2,4-D and 0.5 mgL^−1^ CPPU, 16.25 and 31.75 explants produced **ec** in the 1st and 2nd cycle, respectively (Figure 7b). In both cases, this increase was concomitant with a decrease in the number of explants forming only **nec** (Figure 7a,b).

The formation of **se**, as another parameter of the embryogenic capacity of the explants, also did not change much over the cycles, since nearly all of the explants formed **se** on both types of media (Figure 7c,d). On the lower PGRs concentration, there was a slight (albeit statistically significant) increase in the number of explants forming **se** in the 3rd cycle, as compared to the first two cycles (Figure 7c), whereas on higher concentration, nearly all of the explants developed **se** in both cycles (Figure 7d). However, the number of fully-developed embryos at the cotyledonary stage drastically decreased with the cycles’ progression at both lower and higher concentrations of PGRs. Thus, the number of explants with developed **cse** decreased from 25 in the 1st to 7.5 in the 3rd cycle, and from 22.5 in the 1st to only 2.25 in the 2nd cycle on lower and higher concentrations, respectively (Figure 7c,d). Considering only explants that did form **cse**, the average number of newly-formed **cse** per explant was not significantly different among different SE cycles and different concentrations, being ≈3 in all cases (data not shown). This means that in the 2nd cycle at the higher concentration, less than 10 **cse** were formed, which was insufficient to initiate the 3rd cycle. Overall, 432 **cse** that were used as explants on the lower concentrations (4 Petri dishes x 36 explants/replicate x 3 cycles) produced a total of 634 newly-formed, well-developed **cse** in all cycles, whereas 288 **cse** cultivated on higher PGRs concentrations (4 replicates x 36 explants x 2 cycles) formed a total of 280 **cse**. Most of the **cse** were formed by direct pathway on both media and in all cycles (Figure 7c,d).

### 2.6. Germination of Secondary Somatic Embryos

**Figure 8:**
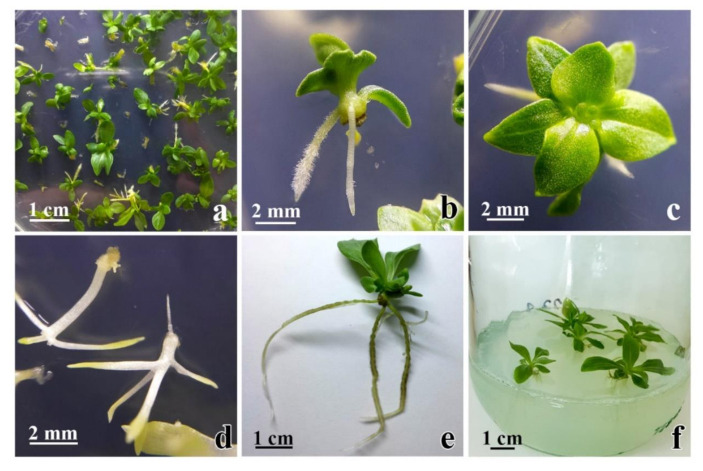
Germination of secondary **cse** on media without PGRs. (**a**–**c**) Most of secondary **cse** germinated in the light had well-developed shoots and some of them formed roots; (**d**) when the secondary **cse** are set to germinate in the darkness, roots and etiolated shoots are formed; (**e**) 40 days old plantlet with well-developed roots; (**f**) plantlets ready for transfer to MS for further development.

The ability of secondary **cse**, obtained on media with 0.1 mgL^−1^ 2,4-D and 0.25 mgL^−1^ CPPU, to germinate and develop into plantlets was tested in the light and in darkness on media without growth regulators. In the light, well-developed shoots with multiple leaves were formed on 80% of the explants (Figure 8a–c), while 32.4% of these plantlets developed roots after 25 days of cultivation (Figure 8b,c). Well-developed and rooted plantlets (Figure 8e) further developed into plants upon transfer to fresh media (Figure 8f). Most plants appeared to be healthy and to grow vigorously. In darkness, 64.5% of the **cse** explants developed etiolated shoots (Figure 8d), and most of those shoots also formed roots.

## 3. Discussion

One of the most important factors in SE induction in most plant species is the concentration of auxins and cytokinins present in the medium. Regarding the requirements for PGRs for the induction of secondary SE, Raemakers et al. [18] concluded that the kinds of PGRs suitable for primary SE were generally suitable for secondary SE as well, and our results corroborate this conclusion. Namely, 2,4-D and CPPU successfully induced both primary [13] and secondary SE in *C. erythraea* (Figure 2, Figure 3, Figure 4 and Figure 5). Similarly, 2,4-D induced both primary and secondary SE in peanut [48] and *Magnolia dealbata* [49], while in some species, hormone-free medium was suitable for efficient induction of both primary and secondary SE [33]. However, there are cases where primary SE is induced by PGRs, but the induction of the secondary SE requires a medium without PGRs for its completion. For example, in carnation, primary ISE was induced through application of 2,4-D and CPPU, while secondary **se** were produced on hormone-free medium [50]. The combination of 2,4-D and CPPU has been investigated during the induction of primary SE [13,29,50,51,52], but very rarely in the induction of the secondary SE [52,53,54]. Adventitious (secondary) embryos were formed on 2,4-D and CPPU-containing media in grapevine [53] and *Epipremnum aureum* [52], but this combination of PGRs did not induce secondary **se** in peanut [54].

Another important factor for the efficiency of secondary SE induction is developmental stage of primary embryos. Some protocols for successful secondary SE involved the use of **cse** as explants, for example in *P. menziesii* [34] and *A. trifoliata* [35]. Globular **se** were more suitable for inducing secondary SE than **cse** in some plant species. However, efficient secondary SE was recorded for all developmental stages of somatic embryos (heart, torpedo, cotyledonary) in cabbage and cauliflower [55] and *H. nobilis* [33]. Centaury leaf-derived **cse** exhibited great embryogenic potential in our research, since secondary SE occurred on all tested media (Figure 5b).

The pattern and frequency of secondary embryogenesis, as well as callogenesis, on centaury primary **cse** explants depended on the culture medium composition, where combinations of 2,4-D and CPPU and 2,4-D alone were used to examine the embryogenic capacity of the primary **cse**. Callus formation was observed on all media, but there were obvious differences in the frequency and characteristics of the induced calli among different PGRs combinations and concentrations. Nonembryogenic calli (**nec**) were formed on all types of media (Figure 5a). The highest number of explants forming **nec** only was observed on media supplemented with 2,4-D solely, on media with equal levels of 2,4-D and CPPU, and on media with 0.1 mgL^−1^ 2,4-D and 0.25 mgL^−1^ CPPU (Figure 5a). Further increase of CPPU concentration promoted the formation of **ec** on centaury **cse**, and the highest number of explants with **ec** was obtained on medium with the highest CPPU concentration (Figure 5a). To the best of our knowledge, there are no data concerning the effect of increasing concentrations of CPPU on **ec** formation during secondary SE. During primary SE, the increase in CPPU concentrations resulted in **ec** induction, for example, in carnation [50].

In the absence of PGRs and on the media containing 2,4-D only, somatic embryos were formed only directly (Figure 5b and Figure 6a). Nevertheless, DSE was the predominant developmental pathway even on media containing CPPU (compare “**dcse** only” and “**icse** only” graphs, Figure 5b). Our results are consistent with literature, since in almost all plant species, the origin of embryos in secondary SE is direct, regardless of the PGRs used. Direct secondary SE was induced on hormone-free medium [35], on medium with different cytokinins [56], or on medium with 2,4-D [38]. However, in the present study, along with secondary DSE (Figure 3g,h and Figure 5b), secondary ISE also occurred, since the formation of **ec** (Figure 3c–e and Figure 5a) and **icse** on **cse** (Figure 3f,h and Figure 5b) was observed upon the addition of CPPU in the presence of 2,4-D. Two different pathways—DSE and ISE—on the same **se** of *Castanea sativa* during secondary SE were also observed [57]. The importance of explant type and PGRs in culture medium on the induction of SE in centaury are reviewed in the accompanying article [14]. While in centaury root culture, SE manifests by direct pattern on media without growth regulators [43], SE in leaf culture occurs by indirect pattern and is induced by 2,4-D and CPPU [13]. Current results indicate that the morphogenic response of centaury **cse** is complex and that it could be modulated with different PGRs combinations. For the first time, both DSE and ISE were observed on the same explant in centaury in vitro culture.

Although **se** and **cse** were formed on all media used in this study, the highest number of **cse** per explant (Table 1) and the highest number of explants forming **se** or **cse** (Figure 5b) were obtained on medium with the highest CPPU concentration. This finding is in agreement with Chen and Hong [55], who found that increasing concentrations of a urea-type cytokinin, thidiazuron, significantly enhanced the percentages of secondary SE in *Oncidium* cultivars Gower Ramsey and Sweet Sugar. On the contrary, Szewczyk-Taranek and Pawłowska [33] reported that the highest number of **cse** per explant was detected on medium without PGRs, while the increasing concentrations of cytokinins reduced the frequency of secondary SE. CPPU is a highly active diphenylurea-derived cytokinin with efficiency in the induction of various morphogenetic processes, including SE. Our results showed that the secondary embryo formation occurred at low efficiency on hormone-free medium or medium containing only 2,4-D. In *Tetrapleura tetraptera* [58] and *M. dealbata* [49], secondary **se** were formed on the media supplemented with 2,4-D only.

Histological analysis confirmed that secondary **se** formed both directly (Figure 4d–f) and indirectly (Figure 4g–i) on the primary **cse** explants. Histological examination showed that DSE and ISE originated from the **cse** subepidermal cells (Figure 4d,g), indicating multicellular origin of centaury secondary somatic embryos. Generally, the pathway and the onset of SE are determined by the physiological and morphological characteristics of the plant tissue source from which the explant derived [59]. Induction of DSE is restricted to somatic cells of explants which have acquired embryogenic competence [15]. According to Puigderrajols et al. [60], **se** development in cork oak actually begins when epidermal and subepidermal cell dedifferentiation starts and the entire meristematic proliferation is a PEM.

Hereby, we have provided the structural evidence for the multicellular origin of secondary **se** in *C. erythraea*. We have demonstrated that the secondary **se** of centaury may develop from PEM clusters of rapidly dividing cells of subepidermal layer. Such response may be a consequence of the various degrees of cell maturity of **cse** explants and different content of endogenous hormones [61]. In *C. erythraea*, intensive divisions of the cells in the subepidermal layers of **cse** explants, and the capability of many neighboring cells to act in a coordinated manner, led to the differentiation and efficient embryo development. We speculate that this may be the main reason why secondary somatic embryo formation via the multicellular pathway occurs very quickly. Our results indicate that primary and secondary SE in centaury result from two distinct ontogenetic pathways, DSE and ISE. These two processes led to the production of **cse** and the maintenance of embryogenic competence for more than 3 months. Secondary embryos arose directly from the primary **cse** embryos, where some epidermal and/or subepidermal cells may have already been embryogenically determined [62]. Regarding secondary ISE, the formation of **se** from the **ec** suggested that cotyledon cells divided and proliferated before some of the callus cells had reached embryogenic competence; thus, a callogenesis stage occurred prior to initiation of the embryogenic process.

The process of centaury secondary SE is asynchronous (Figure 3e,h) which is in accordance with secondary SE of other species [32,38]. Asynchronous development of **se** in centaury has been found both during DSE from roots [43] and ISE from leaf explants [13]. Secondary embryos originated from the entire surface of the centaury primary **cse** (Figure 3c,e and Figure 4a–c). In other species, secondary **se** are formed from different parts of primary **se,** for example, from cotyledons [38], hypocotyls [63,64], hypocotyl/root zone [57], roots [65], combinations of these organs, for example, from cotyledons and radicles [66], or stomatal guard cells [56].

The induction of secondary SE in centaury on media containing 0.5 mgL^−1^ CPPU produced **se** with abnormal morphology (Figure 6c,d). Different causes have been proposed for the abnormality in **se** development, such as excessive PGR addition, long exposition, or accumulation of exogenous auxins inside the tissue [67]. Secondary **se** with abnormal morphology could be reused for callus reinduction, as proposed by Ji et al. [37]. Abnormal morphology of secondary embryos could affect their germination and conversion to plantlets. As reported by Ji et al. [37], the best conversion rate had mono- and bi-cotyledonary embryos (about 60%), followed by poly-cotyledonary and trumpet-shaped embryos. Because of this, embryos of abnormal morphology are usually discarded; however, some of them could also be recycled to induce cyclic SE. The response to hormonal treatment can be dependent on the shape of the **cse**, and only fused cotyledonary embryos were successfully induced into **ec** [37]. Maturation and conversion of somatic embryos into plantlets are important processes which enable the establishment of efficient regeneration systems [68]. In the present study, well-developed bi-cotyledonary **cse** were produced predominantly on media containing 0.1 mgL^−1^ 2,4-D and 0.25 mgL^−1^ CPPU and, therefore, these **se** were transferred on media without growth regulators for germination and conversion (Figure 8a–d). Upon transferring, healthy plants were obtained (Figure 8e,f).

Secondary embryogenesis offers the possibility for enhanced production of somatic embryos through establishment of cycling cultures; thus, it is of great importance to determine specific conditions under which cyclic SE occurs [68]. In this study we investigated the effects of two combinations of 2,4-D and CPPU on embryogenic response of **cse** across several cycles (Figure 1 and Figure 7). We found that the embryogenic potential (production of **ec** and **se**) of centaury **cse** explants increased with the cycles’ progression. Centaury **cse** showed a high rate of embryogenic callogenesis, which increased with cycles’ progression on both media (Figure 7a,b), while the production of **se** increased in the 3rd cycle on lower concentration (Figure 7c), and nearly all explants produced **se** on the higher concentration during two cycles (Figure 7d). Literature encompassing cyclic SE shows that the efficiency of cyclic embryogenesis varies among different species. In *Musa acuminata* AAA cv. Grand Naine, the potential of the explants to produce **se** did not decline with the number of cycles [69]. The embryogenic potential and the mean number of embryos per explant displayed a gradual reduction with subculturing in *A. trifoliata* [35]. Similarly, the percentage of explants with **se** decreased with each cycle of SE induction in *M. dealbata* [49] and in two *Brassica oleracea* varieties [55]. In *C. persicum,* embryogenic competence of calli was affected by number of subculture cycles, since calli from the first cycle showed the highest competence for SE, which decreased during second subculture [32]. Pires et al. [36] developed strategy for recovering and maintaining the cyclic embryogenesis in olive embryogenic calli by its subculturing, which increased the average number of **se** per calli. Therefore, **ec** produced in centaury cyclic embryogenic system could be recycled by transferring onto medium which could enhance maturation of **icse**.

In the present study, the number of explants producing **cse** decreased on both types of media during the cycles (Figure 7c,d). The observed discrepancy in the number of the explants forming **se** of any developmental stage and the number of explants with developed **se** at the cotyledonary stage means that the embryogenic potential of the explants did not decrease, but that the rate of the embryo maturation slowed down with subculturing. Decline in the number of fully-developed **cse** observed in centaury cyclic SE could represent an evidence that long-term maintenance on inductive media affected somatic embryo capacity to advance to later stages. Long exposition to auxin 2.4-D could affect embryo maturation, even though this auxin was proved to be an important factor in the SE induction. Some studies have shown that the presence of 2,4-D in medium is conducive to **ec** induction and proliferation, but that the reduction or removal of 2,4-D promotes **se** development and maturation [38,70]. The **ec** obtained from the immature zygotic embryo of pine trees could produce **se** [71], but the **se** production rate was low, and maturation of **se** was limited. Prolonged culture on the induction medium resulted in an increase in the number of globular and heart-shaped embryos, but did not stimulate the production of mature embryos [64]. In addition, **ec** may lose the potential for SE after extended subculture on medium supplemented with 2,4-D. Although cyclic SE was initiated in both 2,4-D and CPPU combinations in this study, the number of cycles was affected by the higher concentrations of 2,4-D and CPPU. We can speculate that the exposition of **cse**, even to low 2,4-D concentration of 0.1 mgL^−1^, during cycles disturbed the balance of endogenous phytohormones in the embryogenic explants and delayed the maturation of newly-produced **cse.**

In this study, secondary SE was reported for the first time in centaury. This new morphogenetic response could provide a long-term source of **ec** and **se** by establishing cycling cultures. Complex morphogenic response of centaury **cse** could be modulated with different PGRs combinations. Cyclic SE obtained in this study could be used in centaury as a method for obtaining an amplified pool of SE tissues and especially **cse**, which can germinate into plants with good development characteristics. The developed secondary and cyclic SE system could also have fundamental merit because it allows for biochemical and molecular comparison of **se** obtained from roots, leaves, and primary **cse** explants in centaury.

## 4. Materials and Methods

### 4.1. Plant Material

*C. erythraea in vitro* culture was established as described previously [13]. Commercial seeds (Jelitto Staudensamen GmbH, Schwarmstedt, Germany) were surface-sterilized with bleach (4% hypochlorite) and germinated on ½ MS medium [72] half-strength salts and vitamins, containing 30 gL^−1^ sucrose and solidified with 7 gL^−1^ agar (Torlak, Beograd, Serbia). Seedlings were transferred to the same medium for further growth. All of the cultures were maintained under a 16/8-h (light/dark) photoperiod at irradiance of 47 μmol m^−2^ s^−1^ and temperature of 25 ± 2 °C.

### 4.2. Induction of Primary SE

Primary SE was induced according to Filipović et al. [13] with slight modifications. Leaf segments were dissected from well-developed, two-month-old in vitro grown plants and cultured, abaxial side down, in Petri dishes containing basal medium formulation consisting of MS salts and vitamins and supplemented with 30 gL^−1^ sucrose, 7 gL^−1^ agar, 0.1 mgL^−1^ 2,4-D (Sigma-Aldrich, Steinheim, Germany) and 0.25 mgL^−1^ CPPU (Sigma-Aldrich, Germany). The leaf explants were maintained in darkness, at temperature of 25 ± 2 °C, and **se** were developed during three weeks. The obtained primary **se** at the cotyledonary stage (**cse**) were used as explants for the induction of secondary and cyclic SE. In parallel, primary SE was also induced on higher PGRs concentrations of 0.2 mgL^−1^ 2,4-D and 0.5 mgL^−1^ CPPU, and the **cse** obtained on this concentration were used in the experiment with the induction of cyclic SE (Figure 1).

### 4.3. Induction of Secondary SE

Primary **cse** obtained on the medium with 0.1 mgL^−1^ 2,4-D and 0.25 mgL^−1^ CPPU were transferred to five different media: a hormone-free MS medium, and media containing 0.1 mgL^−1^ 2,4-D and CPPU at increasing concentrations (0, 0.1, 0.25, and 0.5 mgL^−1^, see Figure 1). The cultures were kept in constant darkness at 25 ± 2 °C. The experiment was performed in seven replicates (Petri dishes) per treatment, with 36 **cse** per replicate. The **cse** explants were systematically arranged in 6 × 6 arrays, numbered, and documented both photographically and by observing under a binocular microscope (Leica WILD, MPS 28/32, M3Z, Wetzlar, Germany). Developmental parameters, such as the number of explants forming calli or secondary embryos and the number of secondary **cse** developed per explant, were recorded after four weeks.

### 4.4. Induction of Cyclic SE

For the induction of cyclic SE, primary **cse**, obtained from leaf explants on medium containing lower (0.1 mgL^−1^ 2,4-D and 0.25 mgL^−1^ CPPU) or higher concentration of PGRs (0.2 mgL^−1^ 2,4-D and 0.5 mgL^−1^ CPPU), were dissected and transferred to the same medium. The cultures were kept in constant darkness at 25 ± 2 °C. In the 1st cycle, secondary **cse** that developed on either of the two media were transferred to fresh media maintaining the same treatment (Figure 1). In the 2nd cycle, tertiary **cse** that formed after four weeks were subcultured on the same fresh media for the 3rd and last cycle. For both lower and higher hormone treatments, the experiment was performed in four replicates with 36 **cse** explants per Petri dish. After four weeks in each cycle, developmental parameters (as in Section 4.3) were recorded.

### 4.5. Germination of Somatic Embryos

The viability of secondary **cse** obtained on medium containing 0.1 mgL^−1^ 2,4-D and 0.25 mgL^−1^ CPPU was evaluated in terms of somatic embryo germination and plantlet conversion. White opaque secondary **cse**, 1.5–2.5 mm in length, were excised from the primary **cse** explants and set to germinate on MS media without PGRs. The germination experiment was conducted in the light with 105 secondary **cse** and in darkness with 75 **cse**. The germination was scored after 25 days in culture as the percentage of **cse** developing shoots and/or roots.

### 4.6. Histological Analysis

Ontogeny of secondary somatic embryos obtained on medium with 0.1 mgL^−1^ 2,4-D and 0.25 mgL^−1^ CPPU was studied by histological analysis. To confirm histologically that secondary SE was indeed induced in the dark, primary **cse** explants obtained on this medium were transferred to the same medium, kept in the dark, and sampled after three weeks. For histological analysis, the **cse** explants, along with developing **se**, were fixed in mixture of formalin–glacial acetic acid–70% ethanol (FAA) for 24 h, dehydrated in an ethanol series, and embedded in Histowax (Histolab, Västra Frölunda, Sweden) at 56–58 °C. Five-μm-thick sections were cut using a Reichter rotary microtome (Reichter, Wien, Austria) and stained with haematoxylin [73]. The sections were observed and photographed under appropriate magnifications using Nikon Eclipse E100 light research microscope (Nikon, Tokyo, Japan). All images were recorded with Bresser MikroCam SP 5.1 camera and software (Bresser, Rhede, Germany).

### 4.7. Data Collecting and Statistical Analysis

The processes of secondary SE on five different media, as well as cyclic SE on two different media, through the three cycles were evaluated by scoring several developmental parameters (events) after four weeks in culture. The scored developmental parameters included the number of **cse** explants that developed: any type of calli, embryogenic calli (**ec**) or exclusively nonembryogenic calli (**nec**), as well as the number of **cse** explants that developed: secondary somatic embryos at any stage or origin (**se**), secondary somatic embryos at the cotyledonary stage (**cse**), and specifically **cse** formed by direct (**dcse**) or indirect path (**icse**). All statistical analyses were performed using the R programming language for statistical computing [74].

The effect of PGRs concentration on the occurrence of specific developmental events on the explants was analyzed with logistic regression using quasi-binomial distribution to account for overdispersion and logit link function. Medium formulations where no explants induced specific types of calli or embryos were not included in the logistic regression models. The statistical significance of the effect of medium formulation was evaluated using likelihood ratio tests, and for parameters where the effect was significant (*p* < 0.05), pairwise comparisons were performed using the emmeans R package [75]. To account for multiple comparisons, Bonferroni correction was applied, and adjusted *p*-values < 0.05 were considered statistically significant. Bar height on graphs represents fitted values for logistic regression models and error bars represent 95% confidence intervals. Statistically significant differences are denoted with a compact letter display on the figures. Average number of secondary **cse** per experimental replicate was analyzed using ANOVA with Tukey post hoc test for pairwise comparisons.

Data from the cyclic SE experiment were analyzed using generalized estimating equations, as implemented in R package geepack [76]. This approach was chosen since the observations between the cycles were not independent: **cse** obtained in the replicate (Petri dish) no. 1 of the 1st cycle were used as explants in the replicate no. 1 of the 2nd cycle etc., maintaining the same treatment. Occurrence of specific types of calli or embryos on the explants was analyzed using logistic generalized estimating equations, while average number of secondary **cse** per experimental replicate was analyzed using generalized estimating equations. The statistical significance of the effect of SE cycle was evaluated by Wald test statistic [76], and for parameters where the effect was significant (*p* < 0.05), pairwise comparisons were performed using the emmeans R package [75]. To account for multiple comparisons, Bonferroni correction was applied, and adjusted *p*-values < 0.05 were considered statistically significant. Bar height on graphs represents fitted values for general estimation equations (GEE) logistic regression models and error bars represent 95% confidence intervals. Statistically significant differences are denoted with a compact letter display in figures.

## Figures and Tables

**Table 1:** The effect of media formulation on the average number of secondary **cse** per primary explant. Only explants that formed **cse** were considered. Mean values ± SD per treatment are presented. Different letters denote significant differences (*p* < 0.05) according to Tukey post-hoc pairwise comparisons.

2,4-D [mg l^−1^]	CPPU [mg l^−1^]	cse Per Explant
0	0	1.43 ± 0.44 ^a^
0.1	0	1.18 ± 0.37 ^a^
0.1	0.1	1.61 ± 0.54 ^a^
0.1	0.25	3.28 ± 0.71 ^b^
0.1	0.5	3.87 ± 0.50 ^b^

## Data Availability

Representations of the data are contained in the article, raw data is available upon request.

## References

[1] Schouppe D., Rein B., Vallejo-Marin M., Jacquemyn H. (2017). Geographic variation in floral traits and the capacity of autonomous selfing across allopatric and sympatric populations of two closely related *Centaurium* species. Sci. Rep..

[2] Schat H., Ouborg J., de Wit R. (1989). Life history and plant architecture: Size-dependent reproductive allocation in annual and biennial *Centaurium* species. Acta Bot. Neerl..

[3] Tuluce Y., Ozkol H., Koyuncu I., Ine H. (2011). Gastroprotective effect of small centaury (*Centaurium erythraea* L.) on aspirin-induced gastric damage in rats. Toxicol. Ind. Health.

[4] Mroueh M., Saab Y., Rizkallah R. (2004). Hepatoprotective activity of *Centaurium erythraea* on acetaminophen-induced hepatotoxicity in rats. Phytother. Res..

[5] Valentao P., Andrade P.B., Silva E., Vicente A., Santos H., Bastos M.L., Seabra R.M. (2002). Methoxylated xanthones in the quality control of small centaury *(**Centaurium erythraea)* flowering tops. J. Agr. Food Chem..

[6] Subotiċ A., Jankoviċ T., Jevremoviċ S., Grubišiċ D., Teixeira da Silva J.A. (2006). Plant Tissue Culture and Secondary Metabolites Productions of *Centaurium erythraea* Rafn., a Medical plant. Floriculture, Ornamental and Plant Biotechnology: Advances and Topical Issues.

[7] Šiler B., Živković S., Banjanac T., Cvetković J., Nestorović-Živković J., Ćirić A., Soković M., Mišić D. (2014). Centauries as underestimated food additives: Antioxidant and antimicrobial potential. Food Chem..

[8] Jovanović O., Radulović N., Stojanović G., Palić R., Zlatković B., Gudžić B. (2009). Chemical composition of the essential oil of *Centaurium erythraea* Rafn (Gentianaceae) from Serbia. J. Essent. Oil Res..

[9] Kirbag S., Zengin F., Kursat M. (2009). Antimicrobial activities of extracts of some plants. Pak. J. Bot..

[10] Đorđević M., Grdović N., Mihailović M., Arambašić-Jovanović J., Uskoković A., Rajić J., Đordjević M., Tolić A., Mišić D., Šiler B. (2017). *Centaurium erythraea* methanol extract protects red blood cells from oxidative damage in streptozotocin-induced diabetic rats. J. Ethnopharmacol..

[11] Hamza N., Berke B., Cheze C., Agli A.N., Robinson P., Gin H., Moore N. (2010). Prevention of type 2 diabetes induced by high fat diet in the C57BL/6J mouse by two medicinal plants used in traditional treatment of diabetes in the east of Algeria. J. Ethnopharmacol..

[12] Đorđević M., Grdović N., Mihailović M., Jovanović J.A., Uskoković A., Rajić J., Sinadinović M., Tolić A., Mišić D., Šiler B. (2019). *Centaurium erythraea* extract improves survival and functionality of pancreatic beta-cells in diabetes through multiple routes of action. J. Ethnopharmacol..

[13] Filipović B.K., Simonović A.D., Trifunović M.M., Dmitrović S.S., Savić J.M., Jevremović S.B., Subotić A.R. (2015). Plant regeneration in leaf culture of *Centaurium erythraea* Rafn. Part 1: The role of antioxidant enzymes. Plant Cell Tissue Organ. Cult..

[14] Simonović A.D., Trifunović-Momčilov M.M., Filipović B.K., Marković M.P., Bogdanović M.D., Subotić A.R. (2021). Somatic Embryogenesis in *Centaurium erythraea* Rafn—Current Status and Perspectives: A Review. Plants.

[15] Quiroz-Figueroa F.R., Rafael R.H., Galaz-Avalos R.M., Loyola-Vargas V.M. (2006). Embryo production through somatic embryogenesis can be used to study cell differentiation in plants. Plant Cell Tissue Organ. Cult..

[16] Karami O., Aghavaisi B., Pour A.M. (2009). Molecular aspects of somatic-to-embryogenic transition in plants. J. Chem. Biol..

[17] Fehér A. (2015). Somatic embryogenesis—Stress-induced remodeling of plant cell fate. Biochim. Biophys. Acta.

[18] Raemakers C.J.J.M., Jacobsen E., Visser R.G.F. (1995). Secondary somatic embryogenesis and applications in plant breeding. Euphytica.

[19] Guan Y., Li S.G., Fan X.F., Su Z.H. (2016). Application of somatic embryogenesis in woody plants. Front. Plant Sci..

[20] Giri C.C., Shyamkumar B., Anjaneyulu C. (2004). Progress in tissue culture, genetic transformation and applications of biotechnology to trees: An overview. Trees.

[21] Engelmann F. (2011). Use of biotechnologies for the conservation of plant biodiversity. Vitro Cell Dev. Biol. Plant.

[22] Ibáñez S., Carneros E., Testillano P.S., Pérez-Pérez J.M. (2020). Advances in Plant Regeneration: Shake, Rattle and Roll. Plants.

[23] Altamura M., Della Rovere F., Fattorini L., D’Angeli S., Falasca G., Germana M., Lambardi M. (2016). Recent Advances on Genetic and Physiological Bases of *In Vitro* Somatic Embryo Formation. In Vitro Embryogenesis in Higher Plants.

[24] Loyola-Vargas V., Ochoa-Alejo N., Loyola-Vargas V., Ochoa-Alejo N. (2016). Somatic embryogenesis. An overview. Somatic Embryogenesis: Fundamental Aspects and Applications.

[25] Tomiczak K., Mikuła A., Niedziela A., Wójcik-Lewandowska A., Domżalska L., Rybczyński J.J. (2019). Somatic embryogenesis in the family Gentianaceae and its biotechnological application. Front. Plant Sci..

[26] Fehér A. (2008). The initiation phase of somatic embryogenesis: What we know and what we don’t. Acta Biol. Szeged..

[27] Jiménez V.M., Bangerth F. (2001). Endogenous hormone levels in explants and in embryogenic and non-embryogenic cultures of carrot. Phys. Plant.

[28] Vondráková Z., Krajňáková J., Fischerová L., Vágner M., Eliášová K., Park Y.S., Bonga J.M., Moon H.K. (2016). Physiology and role of plant growth regulators in somatic embryogenesis. Vegetative Propagation of Forest Trees.

[29] Fiuk A., Rybczynski J.J. (2008). Genotype and plant growth regulator dependent response of somatic embryogenesis from *Gentiana* spp. leaf explants. Vitro Cell Dev. Biol. Plant.

[30] Cantelmo L., Soares B.O., Rocha L.P., Pettinelli J.A., Callado C.H., Mansur E., Casteller A., Gagliardi R.F. (2013). Repetitive somatic embryogenesis from leaves of the medicinal plant *Petiveria alliacea* L.. Plant Cell Tissue Organ. Cult..

[31] Yang X., Lu J., Teixeira da Silva J.M., Ma G. (2012). Somatic embryogenesis and shoot organogenesis from leaf explants of *Primulina tabacum*. Plant Cell Tissue Organ. Cult..

[32] You C.R., Fan T.J., Gong X.Q., Bian F.H., Liang L.K., Qu F.N. (2011). A high-frequency cyclic secondary somatic embryogenesis systemfor *Cyclamen persicum* Mill. Plant Cell Tissue Organ. Cult..

[33] Szewczyk-Taranek B., Pawłowska B. (2015). Recurrent somatic embryogenesis and plant regeneration from seedlings of *Hepatica nobilis* Schreb. Plant Cell Tissue Organ. Cult..

[34] Lelu-Walter M.A., Gautier F., Eliášová K., Sanchez L., Teyssier C., Lomenech A.M., Le Mette C., Hargreaves C., Trontin J.F., Reeves C. (2018). High gellan gum concentration and secondary somatic embryogenesis: Two key factors to improve somatic embryo development in *Pseudotsuga menziesii* [Mirb.]. Plant Cell Tissue Organ. Cult..

[35] Zou S., Yao X., Zhong C., Shuaiyu Z., Xiaohong Y., Caihong Z., Dawei L., Zupeng W., Hongwen H. (2019). Recurrent somatic embryogenesis and development of somatic embryos in *Akebia trifoliata* (Thunb.) Koidz (Lardizabalaceae). Plant Cell Tiss Organ. Cult..

[36] Pires R., Cardoso H., Ribeiro A., Peixe A., Cordeiro A. (2020). Somatic embryogenesis from mature embryos of *Olea europaea* L. cv. ‘Galega Vulgar’ and long-term management of calli morphogenic capacity. Plants.

[37] Ji W., Luo Y., Guo R., Li X., Zhou Q., Ma X., Wang Y. (2017). Abnormal Somatic Embryo Reduction and Recycling in Grapevine Regeneration. J. Plant Growth Regul..

[38] Karami O., Deljou A., Kordestani G.K. (2008). Secondary somatic embryogenesis of carnation (*Dianthus caryophyllus* L.). Plant Cell Tiss. Organ. Cult..

[39] Klimaszewska K., Noceda C., Pelletier G., Label P., Rodriguez R., Lelu-Walter M.-A. (2009). Biological characterization of young and aged embryogenic cultures of *Pinus pinaster* (Ait.). In Vitro Cell Dev. Biol. Plant.

[40] Pila Quinga L.A., Pacheco de Freitas Fraga H., do Nascimento Vieira L., Guerra M.P. (2017). Epigenetics of long-term somatic embryogenesis in *Theobroma cacao* L.: DNA methylation and recovery of embryogenic potential. Plant Cell Tissue Organ. Cult..

[41] Barešová H., Kamínek M., Novák F.J., Havel L., Doležel J. (1984). Light induce embryogenesis in suspension culture of *Centaurium erythraea*. International Symposium Plant Tissue and Cell Culture Application to Crop Improvement.

[42] Subotić A., Budimir S., Grubišić D., Momčilović I. (2003). Direct regeneration of shoots from hairy root cultures of *Centaurium erythraea* inoculated with *Agrobacterium rhizogenes*. Biol. Plantarum..

[43] Subotić A., Grubišić D. (2007). Histological analysis of somatic embryogenesis and adventitious formation from root explants of *Centaurium erythreae* Gillib. Biol. Plant.

[44] Subotić A., Jevremović S., Grubišić D. (2009). Influence of cytokinins on *in vitro* morphogenesis in root cultures of *Centaurium erythraea*—valuable medicinal plant. Sci. Hortic..

[45] Subotić A., Jevremović S., Grubišić D., Janković T., Jain S.M., Saxena P.K. (2009). Spontaneous plant regeneration and production of secondary metabolites from hairy root cultures of *Centaurium erythraea* Rafn. Protocols for In Vitro Cultures and Secondary Metabolite Analysis of Aromatic and Medicinal Plants, Methods in Molecular Biology.

[46] Simonović A.D., Filipović B.K., Trifunović M.M., Malkov S.N., Milinković V.P., Jevremović S.B., Subotić A.R. (2015). Plant regeneration in leaf culture of *Centaurium erythraea* Rafn. Part 2: The role of arabinogalactan proteins. Plant Cell Tissue Organ. Cult..

[47] Ćuković K., Dragićević M., Bogdanović M., Paunović D., Giurato G., Filipović B., Subotić A., Todorović S., Simonović A. (2020). Plant regeneration in leaf culture of *Centaurium erythraea* Rafn. Part 3: De *novo* transcriptome assembly and validation of housekeeping genes for studies of *in vitro* morphogenesis. Plant Cell Tissue Organ. Cult..

[48] Baker C.M., Wetzstein H.Y. (1995). Repetitive somatic embryogenesis in peanut cotyledon cultures by continual exposure to 2,4-D. Plant Cell Tissue Organ. Cult..

[49] Chávez-Cortazar A., Mata-Rosas M., Oyama K., Samain M.S., Quesada M. (2020). Induction of somatic embryogenesis and evaluation of genetic stability in regenerated plants of *Magnolia dealbata*. Biol. Plant.

[50] Aalifar M., Arab M., Aliniaeifard S., Dianati S., Mehrjerdi M.Z., Limpens E., Serek M. (2019). Embryogenesis efficiency and genetic stability of *Dianthus caryophyllus* embryos in response to different light spectra and plant growth regulators. Plant Cell Tissue Organ. Cult..

[51] Fiore S., De Pasquale F., Carimi F., Sajeva M. (2002). Effect of 2,4-D and 4-CPPU on somatic embryogenesis from stigma and style transverse thin cell layers of Citrus. Plant Cell Tissue Organ. Cult..

[52] Zhang Q., Chen J., Henny R.J. (2005). Direct somatic embryogenesis and plant regeneration from leaf, petiole, and stem explants of Golden Pothos. Plant Cell Rep..

[53] Nakano M., Sakakibara T., Watanabe Y., Mii M. (1997). Establishment of embryogenic cultures in several cultivars of *Vitis vinifera* and *V. x labruscana*. Vitis.

[54] Little E.L., Magbanua Z.V., Parrott W.A. (2000). A protocol for repetitive somatic embryogenesis from mature peanut epicotyls. Plant Cell Rep..

[55] Pavlović S., Vinterhalter B., Zdravković-Korać S., Zdravković J., Cvikić D., Mitić N. (2013). Recurrent somatic embryogenesis and plant regeneration from immature zygotic embryos of cabbage (*Brassica oleracea* var. capitata) and cauliflower (*Brassica oleracea* var. botrytis). Plant Cell Tissue Organ. Cult..

[56] Chen J.T., Hong P.I. (2012). Cellular origin and development of secondary somatic embryos in *Oncidium* leaf cultures. Biol. Plant.

[57] Corredoira E., Ballester A., Vieitez A.M. (2003). Proliferation, Maturation and Germination of Castanea sativa Mill. Somatic Embryos Originated from Leaf Explants. Ann. Bot.

[58] Opabode J.T., Akinyemiju O.A., Ayeni O.O. (2011). Plant Regeneration via Somatic Embryogenesis from Immature Leaves in *Tetrapleura tetraptera* (Schum. & Thonn.) Taub. Arch. Biol. Sci..

[59] Gaj M.D. (2004). Factors influencing somatic embryogenesis induction and plant regeneration with particular reference to *Arabidopsis thaliana* (L.) Heynh. Plant Growth Regul..

[60] Puigderrajols P., Mir G., Molinas M. (2001). Ultrastructure of early secondary embryogenesis by multicellular and unicellular pathways in cork oak (*Quercus suber* L.). Ann. Bot.

[61] Grzyb M., Kalandyk A., Mikuła A. (2018). Effect of TIBA, fluridone and salicylic acid on somatic embryogenesis and endogenous hormone and sugar contents in the tree fern *Cyathea delgadii* Sternb. Acta Physiol. Plant.

[62] Williams E., Maheswaran G.G. (1986). Somatic Embryogenesis: Factors Influencing Coordinated Behaviour of Cells as an Embryogenic Group. Ann. Bot..

[63] Fernández-Da Silva R., Hermoso-Gallardo L., Menéndez-Yuffá A. (2005). Primary and secondary somatic embryogenesis in leaf sections and cell suspensions of *Coffea arabica* cv. Catimor. Interciencia.

[64] Chitra Devi B., Narmathabai V. (2011). Somatic embryogenesis in the medicinal legume *Desmodium motorium* (Houtt.) Merr. Plant Cell Tiss Organ. Cult..

[65] Nair R.R., Dutta Gupta S. (2006). High-frequency plant regeneration through cyclic secondary somatic embryogenesis in black pepper (*Piper nigrum* L.). Plant Cell Rep..

[66] Ćalić D., Zdravković-Korać S., Radojević L. (2005). Secondary embryogenesis in androgenic embryo cultures of *Aesculus hippocastanum* L.. Biol. Plant.

[67] Garcia C., de Almeida A.A.F., Costa M., Britto D., Valle R., Royaert S., Marelli J.P. (2019). Abnormalities in somatic embryogenesis caused by 2,4-D: An overview. Plant Cell Tiss Organ. Cult..

[68] Mazri M.A., Naciri R., Belkoura I. (2020). Maturation and Conversion of Somatic Embryos Derived from Seeds of Olive (*Olea europaea* L.) cv. Dahbia: Occurrence of Secondary Embryogenesis and Adventitious Bud Formation. Plants.

[69] Remakanthan A., Menon T.G., Soniya E.V. (2014). Somatic embryogenesis in banana (*Musa acuminata* AAA cv. Grand Naine): Effect of explant and culture conditions. Vitro Cell Dev. Biol. Plant.

[70] Ali M., Mujib A., Tonk D., Zafar N. (2017). Plant regeneration through somatic embryogenesis and genome size analysis of *Coriandrum sativum* L.. Protoplasma.

[71] Gao F., Pen C.X., Wang H., Shen H.L., Yand L. (2020). Selection of culture conditions for callus induction and proliferation by somatic embryogenesis of *Pinus koraiensis*. J. For. Res..

[72] Murashige T., Skoog F. (1962). A revised medium for rapid growth and bioassays with tobacco tissue cultures. Physiol. Plant.

[73] Johansen D.A. (1940). Plant Microtechnique.

[74] R Core Team (2020). R: A Language and Environment for Statistical Computing.

[75] Lenth R. (2020). Emmeans: Estimated Marginal Means, aka Least-Squares Means. https://CRAN.R-project.org/package=emmeans.

[76] Halekoh U., Højsgaard S., Yan J. (2006). The R Package Geepack for Generalized Estimating Equations. J. Stat. Softw..

